# Illuminating Firefly Diversity: Trends, Threats and Conservation Strategies

**DOI:** 10.3390/insects15010071

**Published:** 2024-01-19

**Authors:** Sara M. Lewis, Wan F. A. Jusoh, Anna C. Walker, Candace E. Fallon, Richard Joyce, Vor Yiu

**Affiliations:** 1 IUCN SSC Firefly Specialist Group, Gland, Switzerland; wanf.ajusoh@monash.edu (W.F.A.J.); candace.fallon@xerces.org (C.E.F.);; 2 Department of Biology, Tufts University, Medford, MA 02155, USA; 3 School of Science, Monash University Malaysia, Subang Jaya 47500, Selangor, Malaysia; 4 New Mexico BioPark Society, Albuquerque, NM 87102, USA; 5 The Xerces Society for Invertebrate Conservation, Portland, OR 97232, USA; 6 Hong Kong Entomological Society, Hong Kong, China

**Keywords:** beetles, biodiversity, citizen science, Coleoptera, insects, IUCN Red List, Lampyridae, light pollution, pesticides, tourism

## Abstract

**Simple Summary:**

Fireflies are a fascinating family of bioluminescent beetles with diverse lifestyles and behaviors. This review summarizes new findings on their taxonomic and ecological diversity, then describes recent global efforts to identify and protect the most threatened firefly species. The major threats that can lead to population declines and extinctions include: loss and degradation of the habitats necessary to sustain their entire life cycle; artificial light that disrupts courtship communication; overuse of broad-spectrum insecticides for pest and mosquito control; overtourism; drought and sea level rise caused by climate change. Certain risk factors can help predict which species are likely to be most vulnerable to each threat. The IUCN SSC Firefly Specialist Group is an international effort launched in 2018 to identify which species face the greatest risks of global extinction and to devise strategies to help protect them. So far, standard IUCN Red List criteria have been used to evaluate the conservation status of 150 different fireflies, including the majority of those living in the US and Canada. While these assessments are not a random sample and represent under 7% of global firefly species, about 20% of those assessed to date face heightened extinction risks. Scarcity of information is a problem for many firefly species, although recent surveys and community science projects have contributed valuable new data. The review concludes by describing conservation initiatives underway at local, regional and national levels to protect and manage key habitat for threatened fireflies and identifying high-priority actions for future conservation efforts.

**Abstract:**

Fireflies are a diverse group of bioluminescent beetles belonging to the family Lampyridae. Recent research on their diversity, evolution, behavior and conservation has greatly advanced our scientific understanding of these charismatic insects. In this review, we first summarize new discoveries about their taxonomic and ecological diversity, then focus on recent endeavors to identify and protect threatened fireflies around the world. We outline the main threats linked to recent population declines (habitat loss and degradation, light pollution, pesticide overuse, climate change and tourism) and describe relevant risk factors that predict which species will be particularly vulnerable to these threats. Although global coordination of firefly conservation efforts has begun only recently, considerable progress has already been made. We describe work by the IUCN SSC Firefly Specialist Group to identify species currently facing elevated extinction risks and to devise conservation strategies to protect them. To date, IUCN Red List assessments have been completed for 150 firefly taxa, about 20% of which face heightened extinction risks. The conservation status for many species has yet to be determined due to insufficient information, although targeted surveys and community science projects have contributed valuable new data. Finally, we highlight some examples of successful firefly habitat protection and restoration efforts, and we use the framework of the IUCN SSC Species Conservation Cycle to point out high-priority actions for future firefly conservation efforts.

## 1. Introduction

Fireflies (Coleoptera: Lampyridae) rank among our most charismatic insects, with distinctive bioluminescent courtship displays that make them an excellent flagship group for broader invertebrate conservation efforts. With more than 2200 species worldwide, firefly beetles exhibit surprisingly diverse behaviors and life history traits, including nonluminous day-active adults with pheromone-based courtship, glow-worm fireflies with flightless females, and flashing fireflies whose courtship relies on exchanging bright, species-specific signals [1,2]. Fireflies also inhabit ecologically diverse habitats, including wetlands (e.g., mangroves, rice paddies, marshes, desert seeps), grasslands, forests, agricultural fields, suburban lawns and urban parks. Their predaceous larvae, which can be aquatic, semiaquatic or terrestrial, spend months to years feeding on snails, earthworms and other soft-bodied prey. In contrast, firefly adults are short-lived and typically do not feed. Some taxa are ecological generalists while others have highly specialized habitat and dietary requirements [3]. 

Collectively, fireflies have enriched humanity in myriad ways. In cultures around the world, these bioluminescent insects are held in high esteem; their graceful luminous dances are celebrated in folktales, art, poetry and literature [4]. As a recreational activity, firefly watching offers inspirational, joyful and spiritually uplifting experiences [5]. Firefly luciferase, the first light-producing enzyme to be cloned and genetically sequenced, has provided a powerful tool that enabled many fundamental advances in molecular biology, pharmaceutical and biomedical research [6]. 

Previous reviews have considered many key features of firefly behavior, ecology, biochemistry, evolution and systematics [1,2,7,8,9,10,11,12]. Considerable progress in firefly conservation science and practice has been made during the past two decades, beginning with publication of The Selangor Declaration for the Conservation of Fireflies [13], written by experts attending the Second International Firefly Symposium held in Selangor, Malaysia in August 2010 and subsequently updated in 2014. By 2018, two complementary international organizations had been established to advance scientific research and to foster global firefly conservation efforts. Fireflyers International Network (FIN) supports firefly research, education, advocacy and protection and hosts international symposia where firefly researchers gather to exchange new discoveries and ideas. The Firefly Specialist Group was established by the International Union for Conservation of Nature’s (IUCN) Species Survival Commission with its primary mission being to identify and help conserve threatened firefly species around the world. Specific goals of the Firefly Specialist Group include to: (1) use IUCN Red List criteria to identify firefly species with the greatest extinction risks, (2) identify key threats and conservation issues within each geographic IUCN region, (3) develop and implement conservation actions to protect high-priority species and (4) identify knowledge gaps for future biodiversity research. 

The goal of this review is to provide a broad overview of recent endeavors to identify and conserve at-risk fireflies around the world. First, we summarize new discoveries about lampyrid taxonomic and ecological diversity and then outline the main threats linked to recent population declines (habitat loss and degradation, light pollution, pesticide overuse, climate change and tourism); we also suggest relevant risk factors that can help predict which species will be most vulnerable to various threats. Although global coordination of firefly conservation efforts has only recently begun, we describe work to increase public awareness, present results from Red List assessments that have identified species currently facing elevated extinction risk in various geographical regions and summarize community science projects that may contribute new species occurrence data. Lastly, we offer examples of successful firefly habitat protection and restoration and indicate high-priority areas to focus future conservation efforts. 

## 2. New Contributions to Taxonomic and Ecological Diversity

**Figure 1:**
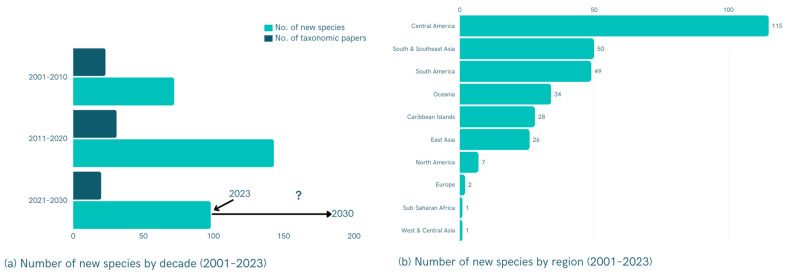
(**a**) Number of taxonomic articles and newly described species of Lampyridae published by decade from 2001 to 2023 (projection to 2030). (**b**) Number of lampyrid species newly described between 2001 and 2023 by geographic region (see Supplementary Table S1 for additional information).

McDermott [14] prepared a comprehensive list of Lampyridae species recorded worldwide; after nearly six decades, this catalog remains a crucial reference for taxonomic studies, although some information may require updating. While the global catalog is undergoing revision, at least 2200 firefly species have now been identified [10]. To summarize taxonomic research conducted on this beetle family over the past two and a half decades, we analyzed publications from 2001 to 2023 using data from Google Scholar through Publish or Perish software v. 7.30 [15]. This analysis revealed 75 taxonomic publications describing a total of 313 new firefly species during this period and an increasing rate of new species discovery (Figure 1a; Supplementary Table S1). However, species discovery rates varied greatly across geographic regions (Figure 1b), likely reflecting the distribution of relevant expertise. Most new species descriptions have come from Central America (Mexico), South and Southeast Asia and South America, with lower rates of species discovery in Europe, North America (United States/Canada) and East Asia. Unfortunately, our understanding of lampyrid diversity in Africa and West and Central Asia remains quite limited, and this represents an important region for future research. About half of all new species belong to the subfamily Lampyrinae and were mainly described from Mexico [16,17] (Supplementary Table S1). Additionally, about one-fourth belong to the subfamily Luciolinae and were described mainly from South and Southeast Asia and Oceania regions [18]. About one-third of the newly described species belong to the genus *Photuris* (Supplementary Table S1). New lampyrid genera include *Oculogryphus* from Vietnam [19], *Memoan* from the Atlantic Forest in Brazil [20] and *Sclerotia* and *Triangulara* from Southeast Asia [21].

Of the 11 recognized subfamilies in Lampyridae [10,22,23], there remain seven genera and the subtribe Vestini that require subfamilial classification as noted by Martin et al. [10]. The current lack of revision presents challenges for identifying various species of common Asian genera. For example, despite the revision of all Luciolinae genera in the past two decades, *Colophotia* and *Curtos* are still awaiting revision [18]. Furthermore, research on genera with flightless females (e.g., *Pyrocoelia*, *Diaphanes*, *Lamprigera*) has largely focused on East Asian populations, and there is still much to uncover about these groups in South and Southeast Asia.

While new species discovery has lagged for certain taxonomic groups within the Lampyridae, recent work has provided a better understanding of these enigmatic groups and has clarified the status of many poorly known firefly species. Some new species discoveries came from major systematic revisions based on the review of type material of Lampyridae, which also prompted changes in other nomenclatural acts: new subfamily, new genera, new synonymies and new combinations. In addition, phylogenetic analyses and other factors have signaled the need for new generic categories; for example, both the Genji firefly (formerly known as *Luciola cruciata*) and the Kumejima firefly (formerly *Luciola owadai*) are now treated under a new genus and combination, *Nipponoluciola* [24]. Furthermore, research using molecular phylogenetics coupled with species delimitation tools has revealed previously unknown cryptic species in *Pteroptyx* fireflies in Malaysian mangroves [25]. This highlights the importance of broadening the scope of ecological, geographical, taxonomic and genetic sampling to better understand the hidden diversity of fireflies.

To complement our expanding knowledge of firefly taxonomic diversity, advances have also been made in understanding the ecological diversity of this beetle family. Although most fireflies are terrestrial during their larval stage, some are aquatic, and metabolic mechanisms enabling such adaptation to freshwater habitats have been explored [26]. In addition, a few fireflies have recently been found living in marine intertidal habitats [27,28], with larvae that are capable of surviving salt water immersion; these include *Micronaspis gabrielae*, a newly described species occurring on rocky coastlines in northeastern Brazil, and two *Atyphella* species inhabiting the rocky intertidal zone on the South Pacific islands of Vanuatu. *Psilocladus costae* is a newly described species from the Atlantic Forest of Brazil [29]; its semiaquatic larvae inhabit epiphytic bromeliads, where they may feed on termites and the aquatic larvae of scirtid beetles. Adding to previous work on the predatory behavior of North American *Photuris* [2,30], new observations reveal that *Photuris lugubris* females hunt and feed on males within mating aggregations of the synchronous firefly *Photinus palaciosi* in central Mexico [31].

Despite these new discoveries, we still know nothing about the larval morphology, larval behavior or larval ecology of an estimated 93% of all known firefly species [32]. This critical knowledge gap hinders our understanding of firefly conservation, especially considering that the larval period constitutes the majority of the firefly life cycle.

## 3. Understanding Firefly Extinction Threats and Risk Factors

While quantitative data on population trends are sparse, detailed monitoring of some well-studied firefly species has revealed significant declines taking place over recent decades (reviewed in [33]). In addition, anecdotal reports and numerous expert opinions suggest reductions in both habitat occupancy and numerical abundance for many firefly species. The major extinction threats faced by fireflies in various geographical regions were reviewed recently [33]; these are briefly described below, together with specific examples that exemplify the ecological and behavioral risk factors predicted to make certain species particularly vulnerable to certain threats [3,34].

### 3.1. Shrinking Spaces: Loss and Degradation of Habitats

Habitat loss and degradation due to human activities continue to be major drivers of biodiversity declines worldwide [35]. As beetles, fireflies undergo complete metamorphosis and thus often utilize distinct habitats initially for larval development and later for adult courtship and mating activity. Those fireflies that are narrow habitat specialists (e.g., wetland species) and species with low dispersal ability (e.g., glow-worms and other species with flightless females) face elevated risks of local extinction due to degradation of their larval habitat, their adult habitat or both, as they are less likely to colonize new sites.

**Figure 2:**
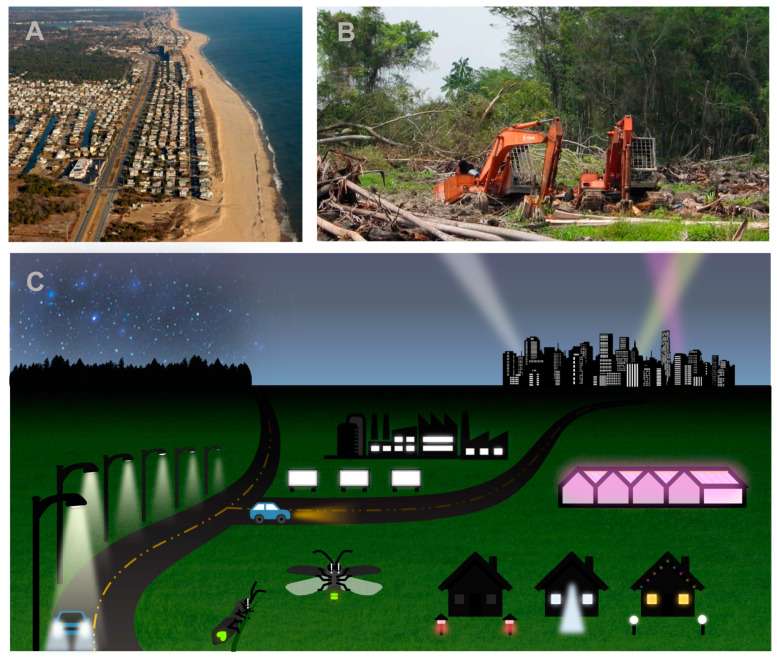
Among the top threats facing fireflies are loss of suitable habitat for firefly juvenile stages and adult reproduction and excessive artificial light at night; both occur on a local scale yet are pervasive globally: (**A**) Residential development, roads and construction have replaced the interdunal wetland habitat of *Photuris bethaniensis* along parts of the mid-Atlantic coast, USA (photo by US Army Corps of Engineers). (**B**) Clearing of riverine mangrove forest in peninsular Malaysia degrades habitat used by *Pteroptyx tener* life stages (photo by Lawrence Kirton). (**C**) Illustration depicting diverse sources of artificial light that can disrupt the nocturnal courtship signaling of fireflies and glow-worms; sources include streetlights, car headlights, commercial and greenhouse lighting, skyglow from cities and residential security, decorative and interior lighting (illustration modified from [48], CC BY).

Many US fireflies occupy freshwater or coastal wetlands, including several species that are classified as threatened on the IUCN Red List due to the loss and degradation of their specialized habitats [36]. One example is the Bethany Beach firefly, *Photuris bethaniensis*, described in 1953 and currently holding the unenviable distinction of being the most critically endangered firefly in the United States [37]. To complete its life cycle, this species relies upon a rare wetland type known as interdunal swales, a freshwater habitat that forms between sand dunes along the mid-Atlantic coast [38]. Within recent decades its unique habitat has succumbed to extensive residential and commercial development (Figure 2A), and the most robust *P. bethaniensis* population was extirpated by the construction of a housing development within the past few years [39]. Despite extensive survey efforts, only a few dozen *P. bethaniensis* populations have been found, and all occurrences are located within state or national parks or wildlife refuges [37].

Agricultural expansion, a major driver of habitat loss globally, also threatens several fireflies. In central west Brazil near Emas National Park, a decline in firefly species diversity was recorded between the 1990s and 2021 following the replacement of the original cerrado and cerradão habitats by extensive soy and sugarcane plantations [40]. 

*Pteroptyx tener* is native to the Southeast Asian region, where it congregates in mangrove trees along riverbanks and emits synchronous flashes that delight thousands of tourists every year [5]. The mangrove habitats upstream of estuaries where this species occurs are being lost to oil palm plantations, subsistence agriculture and aquaculture ponds (Figure 2B) [41]. At one site, the Rembau-Linggi estuary in Malaysia, the mangrove area decreased by 18% from 2002 to 2017 due to replacement by oil palm plantations, rubber plantations, settlements, urban development and roads [42]. Firefly populations at the site subsequently declined [41]. Where agricultural and aquaculture activities are adjacent to mangrove habitats, additional problems may arise due to runoff of insecticides, fertilizers and aquaculture wastes, which further degrade firefly habitats [43,44].

Another firefly threatened by habitat degradation due to agricultural conversion and development is *Nipponoluciola owadai*, an aquatic species endemic to Kume-jima Island in Okinawa Prefecture, Japan [45]. These larvae live in clear, well-oxygenated streams, where they prey on freshwater snails, and adult males flash in synchrony as they fly among the dense riparian vegetation. Development projects on the island have resulted in decreased stream flow and loss of riverside vegetation, and the conversion of traditional rice farming to sugarcane cultivation has caused heavy sedimentation in the streams inhabited by larval *N. owadai* and their snail prey. Although not yet assessed for the global IUCN Red List, this species was described as Critically Endangered on the Japan National Red List. In addition, this species has been recognized as a natural monument of Okinawa Prefecture and listed as an endangered species of wild flora and fauna under the Law for the Protection of Cultural Properties and the Act on Conservation of Endangered Species, respectively [24].

### 3.2. Too Bright: Artificial Light at Night (ALAN)

Light pollution constitutes a known threat to firefly populations worldwide (Figure 2C). Over the past few decades, both the extent and the intensity of artificial light at night have increased exponentially [46]. Light pollution disrupts natural cycles of light and dark and has the potential to mask bioluminescent communication, including the courtship signals that many fireflies require to successfully locate their mates [47]. Nocturnally active fireflies appear to be particularly susceptible to negative impacts from light pollution, perhaps because their bioluminescence evolved under natural darkness, in contrast to dusk-active species that court under higher levels of ambient light [48]. Recent field and lab experiments have confirmed that even low levels of artificial light reduce courtship and mating success of several nocturnal species of North American flashing fireflies, as well as European glow-worms [48].

Many European lampyrids are glow-worm fireflies with flightless females [49]. Such species may face heightened extinction risks from light pollution due to their limited ability to move away from brightly lit habitats. Numerous studies have elucidated the impact of ALAN on the courtship and mating success of the common European glow-worm *Lampyris noctiluca*. *L. noctiluca* adults are found in open grasslands, habitats that augment the visibility of the long-lasting glows that their flightless females produce to attract flying, non-luminescent males [49]. These terrestrial larvae develop for 2–3 years, foraging for snail prey in shrubby areas and hedgerows. Light pollution lowers *L. noctiluca* reproductive success, and studies have provided detailed insight into the mechanisms (reviewed by [48]): artificial light reduces female glow duration, diminishes males’ ability to locate females and reduces overall mating success. A precautionary interpretation suggests light pollution will have similar negative impacts on other *Lampyris* species, although studies are lacking for other glow-worms. 

**Figure 3:**
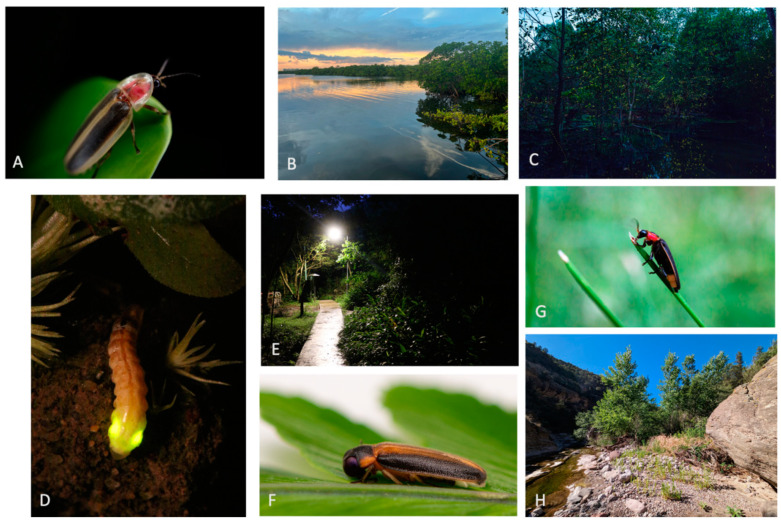
A selection of globally imperiled fireflies in their habitats: (**A**) Female of the threatened Florida intertidal firefly *Micronaspis floridana* (photo by Richard Joyce). (**B**) Mangrove habitat of *Micronaspis floridana*, Florida, USA (photo by Richard Joyce). (**C**) Courtship flashing of *Pteroptyx malaccae* in mangrove forest, Thailand (photo by Banthoon P). (**D**) Courtship glow from a threatened *Oculogryphus chenghoiyanae* flightless female, Hong Kong (photo by Vor Yiu). (**E**) Light pollution impacting the type locality of *Oculogryphus chenghoiyanae* on Lantau Island, Hong Kong (photo by Vor Yiu). (**F**) Male of *Luciola singapura*, listed as Critically Endangered on the 2023 Singapore Red Lists (photo by Shivaram Rasu). (**G**) Male of the threatened Southwest spring firefly *Bicellonycha wickershamorum*, Arizona, USA (photo by Scott Cylwik). (**H**) Perennial stream habitat of *Bicellonycha wickershamorum*, New Mexico, USA (photo by Anna Walker).

*Oculogryphus chenghoiyanae* is a Hong Kong firefly whose flightless females were first described in 2018 [50] and which has been assessed as Critically Endangered on the IUCN Red List (Figure 3). *Oculogryphus chenghoiyanae* occurs along a 1.8 km section of the Fat Mun Ancient Trail on Lantau Island and has not been found elsewhere despite extensive survey efforts. The primary threat to this glow-worm firefly is light pollution produced by the rapidly expanding Hong Kong International Airport and Tung Chung City. At the type locality, the local population disappeared after bright street lamps were erected along the path in 2018 and 2019 [51] (Figure 3E), and a significant decline in female abundance has been observed [52,53]. 

It can be challenging to understand how various threats interact to affect species distribution and abundance. A few recent studies have applied species distribution models to investigate the potential importance of light pollution relative to other threats. One study [54] combined species distribution modeling and threat mapping to examine how changes in light pollution, deforestation and urbanization over the past three decades affected habitat suitability for the tracker ghost *Amydetes fastigiata*, an endemic glow-worm firefly from the Atlantic Forest of Brazil, a biodiversity hotspot [55]. This study identified light pollution as a major emerging threat to fireflies in this region, a pervasive threat that extends even into protected areas. Another study in the Brazilian Atlantic Forest looked at firefly communities along an elevational gradient and used species distribution models to examine how climatological differences interact with light pollution to alter community composition [34]. Climatic variation was found to be the main factor influencing community composition, with low-elevation sites having higher annual mean temperatures that correlated with larger body size and increased abundance of predatory species. In addition, the impact of light pollution differed depending on fireflies’ courtship signaling systems; greater light pollution was associated with decreased abundances of flashing species, yet no association was found between light pollution and abundance of day-active species that use pheromone-based courtship signals. This work confirms that bioluminescent courtship signaling is a key risk factor heightening sensitivity to light pollution and suggests that even within protected areas flashing fireflies will be negatively impacted by artificial light that spills over from cities and other brightly lit areas.

### 3.3. Collateral Damage: Insecticide Overuse

**Figure 4:**
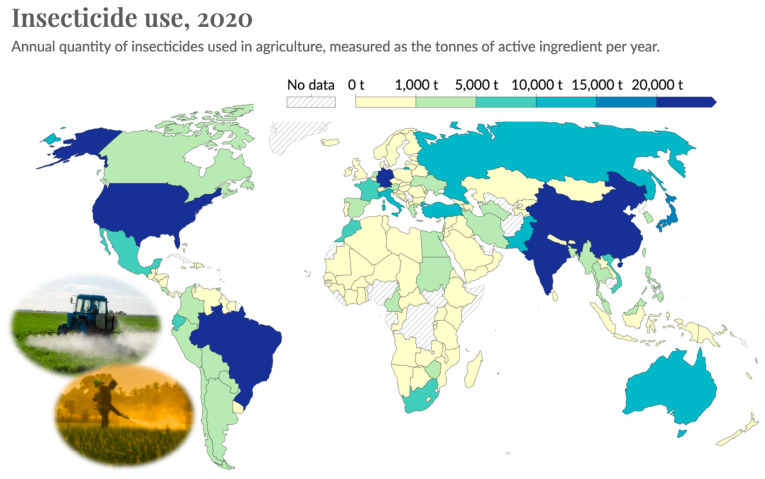
Worldwide use of insecticides in agriculture by country, shown as tonnes of active ingredient applied during 2020. Map published online at OurWorldinData.org (CC BY). Inset photos: Tractor by Aqua Mechanical (Flickr CC BY), farmer photo by Sundaram (Flickr CC BY). For maps showing trends from 1990–2020, see online resource https://ourworldindata.org/grapher/insecticide-use, accessed on 2 January 2024.

Broad-spectrum insecticides, such as organophosphates, carbamates and neonicotinoids, are specifically designed to kill pest insects and are widely used in both agriculture (Figure 4) and residential settings worldwide. However, these compounds also have well-documented lethal and sublethal effects on many non-target insects and other taxa [56,57]. Organophosphate and carbamate insecticides are acetylcholinesterase inhibitors that are extremely toxic to mammals, birds, fish and invertebrates. Although banned in the United States and European Union, the carbamate Furadan is used elsewhere to control soil-dwelling and foliar-feeding pests of crops including rice, soybeans, sugarcane and potatoes. Neonicotinoids, which more narrowly target the nicotinic acetylcholinesterase receptors found in insects and worms, have become the most common class of insecticide used worldwide in both agricultural and residential settings. In the United States, agricultural use of neonicotinoids increased rapidly with the advent of pre-emptive seed treatments for pest management [58]. All of these insecticides can persist for long periods in soils and are readily transported in water. Thus, they can negatively affect diverse organisms inhabiting ecosystems far beyond their application site.

Fireflies are subject to acute or chronic exposure when insecticides are directly applied to the foliage, soil or air in places where fireflies live, when residues are transported through runoff from agricultural or residential applications or when larvae or adults consume pesticide-containing prey. Insecticide residues in water and soil may be particularly harmful because, during their long-lived larval stage, fireflies undergo development for months to years either underwater (aquatic genera such as *Aquatica* and *Sclerotia*) or in soil (terrestrial genera such as *Lampyris*, *Luciola*, *Photinus*, *Photuris*, *Pyrocoelia*). Other firefly life stages may also be exposed as females lay their eggs in soil, moss or rotting wood and as pupae develop underground or on tree trunks. Adults may also be exposed to insecticide residues while resting on treated soil or foliage.

Only a handful of laboratory studies have directly examined pesticide toxicity to fireflies, but these results suggest that chronic exposure to commonly used insecticides can harm fireflies through lethal and sublethal impacts on multiple life stages. Toxicity of several common insecticides (including the neonicotinoid thiamethoxam; the organophosphates acephate, fenthion and diazinon; and others) was tested on the eggs, adults and aquatic larvae of an Asian firefly, *Aquatica lateralis* [59]; full-strength formulations were lethal to these aquatic larvae as well as to adults and also reduced hatching success of eggs. Laboratory studies were recently conducted on the toxicity of the neonicotinoid clothianidin to the larvae of two widely distributed and common North American fireflies, *Photuris versicolor* complex and *Photinus pyralis* [60]. This study found that high soil concentrations of clothianidin reduced the survival of these soil-dwelling larvae. In addition, larvae of both species exhibited marked sublethal responses when exposed to lower soil concentrations, including significantly reduced feeding behavior and impaired movement. Another study [61] tested the neonicotinoid imidacloprid at field-relevant concentrations and found that while it caused low mortality in soil-dwelling larvae of the Asian firefly *Pyrocoelia analis*, its sublethal effects included changes in midgut tissue morphology, convulsive body movements, persistent luminescence and other physiological changes.

These laboratory toxicity studies suggest that insecticides may adversely affect firefly populations, yet field studies are scarce. In a replicated, controlled field test conducted in Maryland, USA, adult firefly abundance showed a 70.4% reduction in plots where corn seeds treated with the neonicotinoid clothianidin had been planted compared with control plots planted with untreated corn [62]. While additional research is needed to determine how various insecticides impact different firefly species and life stages, pesticides can also indirectly harm fireflies by reducing the availability or increasing the toxicity of their larval prey, including earthworms and snails. Imidacloprid and other neonicotinoids are highly toxic to earthworms [57], which constitute a main food source for *Photinus* larvae [4]. Earthworms and other prey also bioaccumulate neonicotinoids [63], representing an additional exposure route for larval fireflies. 

Adult mosquito control typically relies on synthetic pyrethroids, which block neural activity by interfering with voltage-gated sodium ion channels, and these can also harm diverse non-target organisms [64]. No studies have directly examined the impact of various mosquito control methods on fireflies, which may be exposed as pyrethroid sprays are generally applied at dusk when fireflies are active. In field tests, caged ladybeetles (*Harmonia convergens*) exposed to direct contact with permethrin spray showed high acute mortality [65]. Methoprene, an insect growth regulator, is commonly applied to standing water to kill larval mosquitoes; although less harmful to non-target insects compared to other broad-spectrum insecticides, methoprene is toxic to some beetles [66]. Narrow-spectrum insecticides based on bacterial toxins like *Bacillus thuringiensis israelensis* (*Bti*) effectively control mosquito larvae while generally showing minimal toxicity to non-target organisms, including many beetles [67].

### 3.4. Dazzling Light Shows: Overtourism

Fireflies are famous for their mesmerizing bioluminescent displays, and firefly watching has long been a popular summertime activity in Japan [4]. More recently, their remarkable courtship displays have drawn increasing numbers of visitors to sites in the United States, Thailand, Mexico and India; an estimated one million tourists now travel each year to sites in at least 12 countries to watch various firefly species [5]. On the bright side, tourism has the potential to bring economic benefits to local residents and to inspire support for biodiversity conservation. However, unless sites are responsibly managed, tourism can threaten local firefly populations by degrading larval and adult habitats and disrupting adult reproduction. 

Among the species facing the greatest risk from overtourism are various synchronizing fireflies, whose males produce especially stunning displays, and species whose flightless females are highly susceptible to inadvertent trampling when crowds pass through their habitat [3]. *Photinus palaciosi*, a species endemic to forested regions in the mountains of central Mexico, carries both risk factors; it is a synchronous firefly with flightless females. This species was first described in 2012, and since then firefly tourism in Mexico has skyrocketed in popularity, with over 120,000 visitors arriving in 2019 [5]. Such popularity has led to a rapid proliferation of tourism sites, and such a massive influx of tourists has the potential to negatively impact local firefly populations. Irresponsible site management can lead to degraded habitats through soil compaction and excessive light pollution from site infrastructure, vehicles, flashlights, cell phones and cameras. Tourism can also directly impact populations by trampling females and juvenile stages on the ground and disrupting mating pairs that perch on low vegetation.

### 3.5. A Whole New World: Climate Change

Earth’s global climate is changing, bringing more severe and frequent heat waves, drought, storms, wildfires and rising sea levels. Exactly how climate change will impact firefly distributions and abundance remains an open question [68].

Warming temperatures are likely to shift the geographic ranges and phenology of many firefly species. Most fireflies need moisture to complete their life cycle, preventing desiccation of vulnerable immature stages and ensuring availability of their soft-bodied invertebrate prey. Because of their reliance on moisture, fireflies are typically found near permanent water sources. Thus, marked increases in the frequency and severity of drought are likely to be particularly important threats for fireflies in Mediterranean regions and in the arid southwestern United States. One such species is the Southwest spring firefly *Bicellonycha wickershamorum* (Figure 3G,H), which occurs along permanent streams in montane desert habitats in Arizona and New MexicoUSA and Sonora, Mexico, and is threatened by drying up of ephemeral seeps and marshes [69]. At the opposite extreme, flooding and soil erosion associated with more extreme storms and sudden precipitation events have the potential to wash out or transport local populations away from suitable habitats. Climate change has also caused an increase in large, high-intensity wildfires with the potential to destroy firefly populations in areas already stressed by drought, for example, in Canada, southern Europe, and the western United States. In coastal areas, rapid sea level rise threatens to inundate coastal wetlands such as mangroves and salt marshes, with the consequent potential loss of firefly species that require these specialized habitats. 

## 4. Global Firefly Conservation Work

Globally coordinated firefly conservation efforts have only just begun, but considerable progress has already been made. Below, we highlight recent accomplishments in several arenas, including increasing public awareness, using the IUCN Red List to distinguish firefly species with the greatest extinction risk, gathering new data with targeted surveys and community science, protecting threatened fireflies and their habitats and restoring degraded habitats to reintroduce extirpated firefly populations or allow recolonization. 

### 4.1. Increasing Public Awareness 

Effective science communication plays a crucial role in conservation efforts by garnering public support, attracting funding, driving policy initiatives and encouraging informed decision making. In 2018, the Fireflyers International Network and the Malaysia Nature Society promoted the first World Firefly Day, an outreach event to enhance public knowledge about firefly ecology and threats and to build support for invertebrate conservation more broadly. Scheduled annually for the first weekend in July, World Firefly Day now attracts thousands of participants who attend activities such as local firefly-watching festivals, live demonstrations, webinars, art exhibits, night walks and haiku-writing and origami-folding contests. 

The Xerces Society is a science-based organization based in Portland, Oregon, USA, that focuses on the conservation of invertebrates and their habitats. As part of its firefly conservation initiative, Xerces has prepared and distributed science-based guidelines for conserving fireflies in the US and Canada [70]. In response to the increasing popularity of firefly tourism in the US and elsewhere, Xerces has also distributed guidelines outlining best practices for managing tourist sites and visitor etiquette [71,72]. Informational fact sheets on insect-friendly lighting that provide guidance and are aimed at reducing the impact of light pollution on fireflies and other insects are also freely available online [73,74].

### 4.2. Global IUCN Red List: Evaluating Extinction Risks 

The Red List of Threatened Species, published by the International Union for Conservation of Nature (IUCN), is internationally recognized as the most respected and robust inventory of conservation status. Levels of extinction risk for plants, animals and fungi are estimated according to several criteria that use standardized quantitative thresholds to assign each taxon to one of eight categories [75]: Extinct (EX), Extinct in the Wild (EW), Critically Endangered (CR), Endangered (EN), Vulnerable (VU), Near Threatened (NT), Least Concern (LC) and Data Deficient (DD). EX indicates virtual certainty that the last individual of that species has died, and EW means the species persists only in captivity. Threatened species that face either extremely high, very high or high risk of extinction in the wild are assigned to categories CR, EN or VU, respectively, while NT indicates a borderline species that may become threatened in the near future. LC indicates that a species is widespread and abundant or otherwise currently at low risk of extinction, and DD indicates there is still insufficient knowledge of that species to assess extinction risk. The IUCN Red List is a widely used and effective tool for identifying species in greatest need of immediate conservation attention; thus, species categorized as Critically Endangered, Endangered or Vulnerable can be prioritized for conservation efforts at local, regional and national levels. Further, after all the available data are compiled, any species categorized as Data Deficient can be targeted for additional surveys and research.

The IUCN SSC Firefly Specialist Group has been working to determine which of the approximately 2200 firefly species worldwide currently face the greatest risks of extinction. This effort began in 2020 by compiling all available data on species distributions, habitats, life history traits, behaviors and threats for 130 firefly species and 2 subspecies found in the United States and Canada, then determining their conservation status according to the standard Red List criteria [36]. Because most invertebrates typically lack sufficient data on population size or rates of decline [76], these firefly assessments use estimates of each taxon’s distributional range based on existing occurrence data, together with evidence of decline, fragmentation or fluctuation (criterion B) to determine the Red List category. 

**Figure 5:**
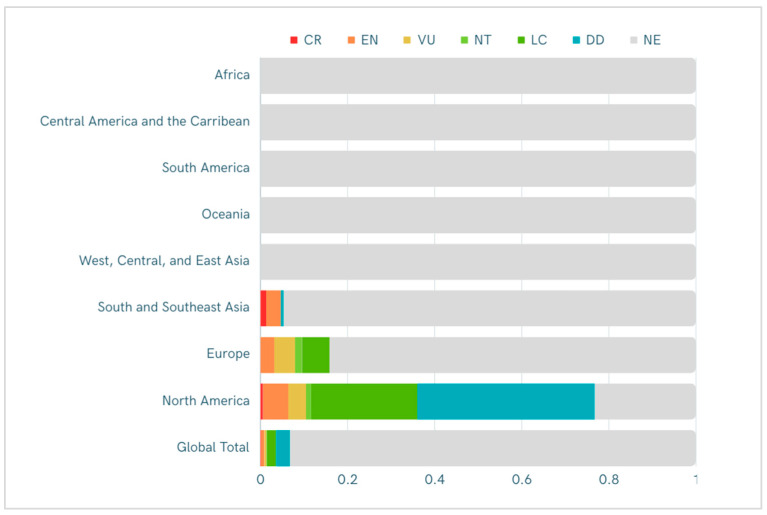
Conservation status of Lampyridae by region, showing the proportion of known species that have been initially evaluated as Critically Endangered (CR), Endangered (EN), Vulnerable (VU), Near Threatened (NT), Least Concern (LC) or Data Deficient (DD) according to IUCN Red List criteria or that have not yet been evaluated (NE). Proportions based on an estimated global total of 2200 species, with estimates of known species numbers for regions as follows: North America (172), Europe (63), South and Southeast Asia (150). (Species numbers not yet estimated for regions lacking any completed assessments).

An analysis of the conservation status for 130 firefly species found in the United States and Canada [36] revealed 18 taxa that are currently categorized as threatened according to these Red List criteria. In addition, approximately half of the assessed species were classified as Data Deficient, while one-third were classified as Least Concern (Figure 5). To date, Red List assessments have also been completed for selected firefly taxa from Europe (10 species), Southeast Asia (4 species) and China (including Hong Kong, 4 species).

While these assessments represent a non-random sample of less than 7% of global firefly species, about 20% of those fireflies that have been assessed to date face heightened extinction risks (Table 1). These threatened taxa include fireflies known or suspected to be restricted to specialized habitats like montane seeps, riparian mangrove forests, salt marshes and cypress swamps; as noted above, such habitat specialists are especially vulnerable to continued habitat loss, degradation and fragmentation.

### 4.3. Community Science Contributions 

Fireflies are compelling subjects, and numerous community science projects have focused on Lampyridae (see Supplementary Table S2). Observations submitted by community scientists in Europe have helped to create and refine firefly species checklists in Spain [77]; Flanders, Belgium [78]; and Croatia [79,80] and have aided in tracking range expansion of the introduced firefly *Photinus signaticollis* through Spain and France [81]. In Hong Kong, a Firefly Survey Team established in 2020 has systematically gathered data on firefly distributions and abundances. Team members are trained by attending eight hours of lectures plus eight hours of guided field practice, and they then conduct surveys for 6–7 months during firefly season. These trained community science volunteers have contributed many new location records for Hong Kong fireflies, have conducted repeated quantitative surveys for several threatened species and have discovered previously undescribed species [52,53]. 

Observations posted to iNaturalist, a photo-based biodiversity platform, have documented previously unmapped species occurrences, including populations of threatened firefly species. In the USA, for example, five of the twenty-five known localities listed in an Endangered Species Act petition for the Florida intertidal firefly *Micronaspis floridana* were documented and discovered based on verified iNaturalist observations [82]. However, many firefly species are difficult or impossible to identify based solely on photos. To be verifiable, observations require additional information such as details of courtship flash behavior and air temperature, and most iNaturalist submissions lack such information. In 2023, the Xerces Society launched a community science project and data portal called Firefly Atlas to crowdsource firefly survey and observation data for the US and Canada. The survey protocol, observation form and record-vetting process of Firefly Atlas help to ensure that records are reliable. 

Using data from community science projects to track the numerical abundance of particular firefly species can be challenging, yet successful examples do exist, including the Hong Kong Firefly Survey described above. In England, transect surveys conducted by citizen scientists of the common European glow-worm *Lampyris noctiluca* at 16 sites showed that counts of glowing females declined 3.5% per year over 18 years [83]. In the Satoyama agroscape of Japan, volunteer citizen scientists have contributed to studies of habitat requirements [84] for the Genji firefly (*Nipponoluciola cruciata*) and collected data on long-term abundance trends [85].

As growing numbers of organizations and individuals participate in firefly community science, efforts should be made to ensure that the data collected are maximally useful for conservation, offering benefits beyond education and outreach. Projects and participants should strive for occurrence records at the lowest taxonomic rank possible, preferably species (or distinct morpho-species cases where taxonomy is not well resolved or species are undescribed). Measurements of abundance or long-term monitoring projects require positive species identification along with collecting basic natural history and seasonality data. This may require a mix of voucher specimens, high-quality voucher photos and detailed notes on behavior, such as courtship flash patterns or glow behavior. When sampling fireflies for presence/non-detection or abundance, incorporating effort metrics and environmental variables such as temperature will facilitate the interpretation of findings [86]. The use of DarwinCore data standards for occurrence records and recording project metadata following standards such as Humboldt Core [87] will help to streamline the sharing and analysis of data collected.

### 4.4. Conservation Actions

In many cases, we already know enough about the ecology, behavior and threats for the most at-risk fireflies to start working with local, regional and national organizations to design conservation strategies that can help mitigate threats, protect habitat and halt population declines. 

In the United States, for example, completed IUCN Red List assessments have already been used to identify which fireflies in each state should be designated as Species of Greatest Conservation Need in State Wildlife Action Plans. However, certain state wildlife agencies lack the authority to protect imperiled insects, so federal regulations are also needed to protect several at-risk firefly species. The strongest and most effective US federal legislation for species conservation is the Endangered Species Act (ESA), a law protecting plant and wildlife species that are listed as threatened or endangered with extinction. Although no firefly species is yet included in the ESA list of protected species, the Critically Endangered Bethany Beach firefly *Photuris bethaniensis* is currently under review by the U.S. Fish and Wildlife Service [39,88]. Furthermore, petitions have been submitted to also list four additional threatened US fireflies: *Micronaspsis floridana* (Figure 3A,B), *Bicellonycha wickershamorum* (Figure 3G,H), *Photuris forresti* and *Photuris mysticalampas* [82,89,90,91]. 

Fireflies already appear on a few National Red Lists. Although National Red Lists may use different criteria than the global IUCN Red List, they provide information about species status within national borders that can be useful for conservation planning. For example, five fireflies are included in the 2023 edition of Singapore Red Lists [92], including *Pteroptyx bearni*, presumed to be nationally extinct, and two critically endangered species: *Pteropytx malaccae* and the recently described *Luciola singapura* (Figure 3F) [93], which was discovered in the last remaining freshwater swamp in Singapore. These Singapore Red Lists, which will be compiled into a third edition of the Singapore Red Data Book, are intended to guide the planning and prioritization of future nature conservation efforts such as habitat enhancement and species recovery programs. 

In China, various categories and levels of protection are applied to certain wild animals. While rare and endangered species are under key protection, the National Forestry and Grassland Administration of China puts other animals that also need protection on a List of Terrestrial Wild Animals of Important Ecological, Scientific and Social Value. Inclusion is based on the joint principles of prioritizing ecological protection, meeting the needs of scientific research and benefiting social development. This list was newly updated in June 2023 [94], and 11 firefly species are amongst the 700 newly added species: *Emeia pseudosauteri*, *Luciola kagiana*, *Sclerotia fui*, *Sclerotia flavida*, *Asymmetricata circumdata*, *Pygoluciola qingyu*, *Aquatica ficta*, *Aquatica leii*, *Aquatica lateralis*, *Pteroptyx maipo* and *Pyrocoelia pectoralis* [95]. 

#### 4.4.1. Habitat Protection

Because habitat loss and degradation are among the most critical threats to fireflies, effective conservation strategies must include not just protecting habitats but also managing them to enable threatened species to complete their entire life cycle. This includes protecting habitat where adult courtship takes place, habitat with suitable oviposition and pupation sites and habitat where the predaceous larvae and their invertebrate prey can thrive. Most threatened fireflies are habitat specialists, therefore sites where they still occur must be actively managed to mitigate known threats and prevent further habitat degradation. Effective management will include not only legal protection for occupied sites but also active management to mitigate light pollution in and around firefly habitat, control mosquito spraying and insecticide use and prevent direct disturbance of vulnerable life stages. 

Throughout Southeast Asia, congregating *Pteroptyx* fireflies are economically important because they attract tourism revenue. In Malaysia, *Pteroptyx tener* males court by flashing in unison while perched on visually prominent trees in riverine mangrove habitats [96]. After mating, females lay eggs in muddy areas adjacent to the river margin, and larvae forage for snails in the intertidal zone. Preliminarily classified as Endangered on the IUCN Red List, *P. tener* faces continuing habitat loss due to conversion of mangrove habitat to oil palm and rubber plantations, urban development, river modification projects and aquaculture farming (Figure 2B). To preserve habitat for firefly tourism along Sungai (River) Selangor, in 2009 the Selangor State Government and the Selangor Water Management Board (LUAS) created a river reserve centered at Kampung Kuantan [97]. This reserve encompasses over 1000 hectares along 40 km of river, including 150–400 m of habitat on either side of the river, and restricts activities such as land clearing. Within the Matang Mangrove Forest Reserve in Perak State, another area has been proposed that would protect firefly habitat along the Sepetang River, but this reserve has not yet been established. Despite existing and planned site level protections, there are currently no conservation plans that are explicitly aimed at protecting suitable habitats for all *P. tener* life stages. To protect populations of this endangered firefly, sites must be actively managed to mitigate known threats and to prevent further habitat degradation. This could include designating a protected buffer zone within the high tide flood line around the rivers’ edge to preserve key larval habitat, reducing runoff from adjacent agricultural lands and aquaculture ponds and preventing further encroachment by development into mangrove forests. Near urbanized areas, light pollution could be limited by planting trees adjacent to firefly habitats or encouraging municipalities to limit artificial light at night. 

The Bethany Beach firefly, *Photuris bethaniensis*, is a Critically Endangered habitat specialist with a restricted distribution along the mid-Atlantic coast of the United States. This species relies on interdunal freshwater swales, a rare wetland habitat type that has disappeared over the past several decades due to increased residential and commercial development (Figure 2A) [37]. All remaining *P. bethaniensis* populations occur within state or federally protected areas, but none of these sites is actively managed to protect fireflies. Effective management plans to protect existing *P. bethaniensis* habitat should include mitigating light pollution from nearby highways and infrastructure, buffering occupied sites from roads and developments and protecting occupied swale sites against disturbance from tourism and other recreational activities. 

Beyond urgently addressing the conservation needs of particular threatened fireflies, protecting certain areas that support high species diversity will help safeguard common fireflies, as well as rarer ones. In the US, an incipient movement is growing among local conservation organizations and land trusts to establish Firefly Sanctuaries, which should be encouraged to follow published guidelines for best management practices [70].

#### 4.4.2. Habitat Restoration and Species Reintroduction

In places where firefly habitat has been partially or largely degraded, restoration projects could restore habitats so they can once again support firefly life cycles in their entirety. While habitat restoration has yet to be attempted for any threatened US fireflies, projects in Asian countries provide good models for successful habitat restoration and species reintroduction, particularly for fireflies with aquatic larvae.

In Japan, the Genji firefly *Nipponoluciola* (formerly *Luciola*) *cruciata* has long been the focal point for summertime firefly-watching activities. By the early 20th century, however, this popular species was in decline due in part to water pollution from increased urbanization and industrial effluent together with river canalization, both of which degraded the river habitat and reduced survival of these aquatic larvae and their snail prey [4]. During the 1970s, many communities around the country undertook municipal projects to clean up rivers and restore suitable habitat. As water quality improved, breeding programs were established and thousands of captive-bred larvae were released into restored rivers. Enthusiastic citizens and schoolchildren joined conservation projects to clean up rivers and to breed and release firefly larvae. The Japanese government designated several areas of high-quality habitats for Genji fireflies as National Natural Monuments, thus establishing legal protection for firefly habitat, and conservation of Genji fireflies still enjoys widespread public support in Japan [98].

In Taiwan, researchers have successfully restored habitat and reintroduced native *Aquatica ficta* fireflies into areas where they were once common but have been extirpated due to urbanization. Fireflies are culturally significant in Taiwan, and although this species is not presently threatened, its aquatic larvae and their snail prey are both easy to breed in captivity. Habitat restoration projects involving collaborations between firefly experts, universities, city officials, community organizations and corporations have successfully reintroduced *A. ficta* into several sites, including Da’an Forest Park in downtown Taipei [99], the Taipei campus of National Taiwan University [100] and several sites owned by the TSMC Corporation, a semiconductor manufacturer [101]. These projects represent proof of concept and can provide protocols for successful habitat restoration and reintroduction that might eventually be adapted to restore extirpated populations of threatened fireflies. 

Even without targeted reintroduction programs, appropriately restored habitats may be colonized from nearby populations. At the southern end of Key Biscayne in Florida, USA, intensive efforts were made during the 1990s to restore mangroves and coastal wetlands to an area previously filled with dredge spoils and surrounded by concrete bulkheads along the shoreline [102]. In 2023, successful colonization of this restored mangrove habitat by the endangered Florida intertidal firefly *Micronaspis floridana* was documented at Bill Baggs Cape Florida State Park (R. Joyce, personal observation).

## 5. Priority Actions for Firefly Conservation

Though many challenges lie ahead, we already know enough to start devising conservation strategies to protect the most at-risk firefly species and to facilitate their recovery. The IUCN Species Survival Commission advocates a strategic approach encapsulated in the Species Conservation Cycle, an iterative process that comprises three sequential pursuits: Assess (compile data to complete Red List assessments, engage in biodiversity discovery, identify key biodiversity areas), Plan (develop conservation plans for threatened species and habitats) and Act (implement conservation plans and policies). Using this framework, below we outline key actions that we believe are the highest priority for future firefly conservation efforts.

### 5.1. Build Partnerships

- Continue public outreach and education to raise awareness of firefly diversity, life cycles and conservation needs.
- Encourage participation in high-quality community science projects that yield verifiable data.
- Seek partnerships to provide sustainable financial support for firefly conservation efforts.
- Build capacity and interest in firefly research and conservation by mentoring students and early career scientists.

### 5.2. Assess

- Expand IUCN Red List assessments across geographic regions to identify which fireflies face greatest the extinction risks and to prioritize the protection of these threatened species.
- Develop, disseminate and implement standardized monitoring protocols to track long-term population trends for threatened fireflies.
- Invest resources in biodiversity exploration in understudied regions, including India, Africa, the Philippines and South and Central America.
- Fill knowledge gaps using species distribution models and other approaches to facilitate research on distribution, habitat use and ecology of threatened firefly taxa.
- Use IUCN Red List criteria to reassess species at regular intervals to track progress and realign conservation priorities.

### 5.3. Plan

- Expand existing protected areas to safeguard resources needed to support all life stages of threatened firefly species (e.g., buffer zones along mangrove rivers).
- Within established protected areas, ensure that conservation needs of threatened firefly species are explicitly included in management plans, for example:
  ○ Reduce light pollution spilling into protected areas.
  ○ Eliminate broad-spectrum insecticide use.
  ○ Redesign mosquito control programs to minimize toxicity to fireflies and other non-target invertebrates.
  ○ Protect fragile wetland and riparian habitat from trampling (e.g., with boardwalks or by fencing sensitive areas off from recreational activities or cattle).

### 5.4. Act

- Restore degraded habitat to provide full life cycle support for threatened fireflies.
- Partner with NGOs and government agencies to secure legal protection for threatened species at state and/or national levels.
- Work with local communities and state, national and regional organizations to implement policies to reduce light pollution and pesticide overuse that threaten fireflies and other insects.
- Collaborate with local organizations and land trusts to designate Firefly Sanctuaries that provide habitat protection and management for threatened species and areas with high species diversity.
- Disseminate guidelines and work with local communities, tour operators and visitors to encourage responsible firefly tourism.

## Figures and Tables

**Table 1:** Current list of fireflies categorized as globally threatened with extinction according to the IUCN Red List criteria, geographic distribution, life history traits and habitat associations.

Species Name	Category	Criteria	Countries of Occurrence	Type of Firefly	Females	Habitat Description
*Bicellonycha wickershamorum* (Southwest spring firefly)	VU	B1ab(iii)	USA, Mexico	Flashing	Winged	Montane seeps and marshes along permanent streams
*Bicellonycha wickershamorum piceum* (Gila Southwest spring firefly)	EN	B2ab(iii)	USA	Flashing	Winged	Montane seeps along permanent streams
*Bicellonycha wickershamorum wickershamorum* (Southwest spring firefly)	VU	B1ab(iii)	USA, Mexico	Flashing	Winged	Montane seeps and marshes along permanent streams
*Lampyris germariensis* (Dalmatian glow-worm)	VU *	B2ab(iii)	Croatia, Montenegro, Bosnia and Herzegovina	Glow-worm	Flightless	Temperate broadleaf and mixed forests
*Lampyris lareynii* (Corsican glow-worm)	VU *	B1ab(iii)	France	Glow-worm	Flightless	Humid microhabitats within Mediterranean woodlands and shrublands
*Lampyris pallida* (Maltese glow-worm)	EN *	B1ab(iii)+2ab(iii)	Malta	Glow-worm	Flightless	Humid microhabitats within Mediterranean shrublands
*Lucidota luteicollis* (Florida scrub dark firefly)	VU	B1ab(iii)	USA	Diurnal	Flightless	Upland ridges within scrub, sandhill, and pine savannah
*Luciola novaki* (Black legged firefly)	EN *	B1ab(iii)+2ab(iii)	Albania, Montenegro	Flashing	Winged	Freshwater and estuarine marshes
*Luciola tuberculata* (Lumpy-necked flasher)	CR	B1ab(iii)+2ab(iii)	Hong Kong	Flashing	Winged	Shrublands and lowland forests along rivers
*Micronaspis floridana* (Florida intertidal firefly)	EN	B2ab(i,ii,iii)	USA, The Bahamas	Flashing	Winged	Salt marshes, mudflats, and mangroves in coastal areas
*Nyctophila calabriae* (Calabrian Nyctophila glow-worm)	EN *	B1ab(iii)	Italy	Glow-worm	Flightless	Riparian areas within woodlands
*Oculogryphus chenghoiyanae* (Chenghoiyan Ototretine firefly)	CR	B1ab(i,ii,iii,v)+2ab(i,ii,iii,v)	Hong Kong	Glow-worm	Flightless	Shrublands and dense, natural woodlands
*Photinus acuminatus* (Pointy-lobed firefly)	EN	B2ab(i,ii,iii,iv,v)	USA	Flashing	Winged	Bogs and marshes
*Photinus knulli* (Southwest synchronous firefly)	VU	B1ab(iii)	USA, Mexico	Flashing	Winged	Marshes along permanent streams
*Photuris bethaniensis* (Bethany Beach firefly)	CR	B1ab(i,ii,iii,v)	USA	Flashing	Winged	Interdunal freshwater swales
*Photuris cinctipennis* (Belted firefly)	EN	B1ab(ii,iii)+2ab(ii,iii)	USA	Flashing	Winged	Moist lowland areas within hardwood forests
*Photuris flavicollis* (Sky island firefly)	VU	B1ab(iii)	USA	Flashing	Winged	Montane seeps and springs
*Photuris forresti* (Loopy five firefly)	EN	B1ab(i,ii,iii,iv)+2ab(i,ii,iii,iv)	USA	Flashing	Winged	Marshes
*Photuris mysticalampas* (Mysterious lantern firefly)	EN	B1ab(ii,iii)+2ab(ii,iii)	USA	Flashing	Winged	Forested peatland floodplains
*Photuris pensylvanica* (Dot-dash firefly)	VU	B2ab(iii)	USA	Flashing	Winged	Tidal and non-tidal freshwater wetlands
*Photuris pyralomima* (None)	EN	B1ab(i,ii,iii)+2ab(i,ii,iii)	USA	Flashing	Winged	Moist grassland or shrubland
*Photuris walldoxeyi* (Cypress firefly)	VU	B2ab(iii)	USA	Flashing	Winged	Cypress swamps
*Pleotomodes needhami* (Ant-loving scrub firefly)	EN	B1ab(iii)	USA	Flashing	Flightless	Upland ridges within xeric pine and oak scrub forests
*Pteroptyx bearni* (The Comtesse’s Firefly)	EN *	B2ab(ii,iii,v)	Malaysia, Singapore (possibly extinct) and Brunei Darussalam (possibly extinct)	Flashing	Winged	Estuarine mangrove forests
*Pteroptyx maipo* (Maipo bent-winged firefly)	EN	B2ab(ii,iii,iv)	Hong Kong	Flashing	Winged	Inter-tidal mudflats and mangrove forests
*Pteroptyx malaccae* (A bent-necked firefly)	EN *	B2ab(iii,v)	Cambodia, Indonesia, Malaysia, Singapore, Thailand	Flashing	Winged	Riverine mangrove forests
*Pteroptyx tener* (A bent-necked firefly)	EN *	B2ab(iii,v)	Indonesia, Malaysia, Thailand	Flashing	Winged	Riverine mangrove forests
*Pteroptyx valida* (A bent-necked firefly)	EN *	B2ab(iii)	Cambodia, Indonesia, Malaysia, Singapore, Thailand	Flashing	Winged	Estuarine mangrove forests
*Pyractomena ecostata* (Keel-necked firefly)	EN	B2ab(i,ii,iii)	USA	Flashing	Winged	Brackish tidal marshes
*Pyractomena vexillaria* (Amber comet)	EN	B2ab(i,iii)	USA, Mexico	Flashing	Winged	River basins within semi-arid shrubland

* Indicates species that have been submitted to the IUCN Red List but have not yet been published; categories and criteria for these species are thus considered preliminary until formal publication.

## Data Availability

Relevant data presented in this review are included within the article or in supplementary material; further inquiries can be directed to the corresponding author.

## Supplementary Materials

The following supporting information can be downloaded at: https://www.mdpi.com/article/10.3390/insects15010071/s1, Supplementary Table S1: List of newly described firefly species from 2001 to 2023, including species counts by subfamily [8,16,17,18,19,20,21,22,27,28,29,50,93,96,103,104,105,106,107,108,109,110,111,112,113,114,115,116,117,118,119,120,121,122,123,124,125,126,127,128,129,130,131,132,133,134,135,136,137,138,139,140,141,142,143,144,145,146,147,148,149,150,151,152,153,154,155,156,157,158,159,160,161,162,163,164,165]. Supplementary Table S2: Community science projects for fireflies.
