# Supplementary material for: Illuminating Firefly Diversity: Trends, Threats and Conservation Strategies
Source: Insects. 2024 Jan 19;15(1):71. doi: 10.3390/insects15010071 (PMC10815995; doi:10.3390/insects15010071)
Supplement: Supplementary file 1 [file insects-15-00071-s001.zip › Supplementary Table S2 Community Science Projects.pdf]

Table 2. List of firefly citizen science and monitoring projects, presented in chronological order of establishment and displaying types of data collected and taxonomic and geographic scope. Single asterisk (*) indicates that some species-level occurrence records may be generated, but it is not a primary goal of the project. Double asterisks (**) indicate that count data are collected but effort metrics are not.

	Type of data generated				**Scope**	
Project	**Species occurrence records**	**Spatial**	**Temporal**	**Abundance**	**Geographic scope**	**Taxonomic scope**
**UK Glow-worm Survey [1]**	Yes	Yes	Yes	Yes	United Kingdom	*Lampyris noctiluca* and other glow-worm spp.
**Natuurpunt Glow-worm workgroup [2]**	Yes	Yes	Yes	Yes**	Belgium	Glow-worm Lampyridae
**Satoyama Firefly Monitoring [3]**	Yes	Yes	Yes	Yes	Satochi-Satoyama agroscape of Japan	*Nipponluciola cruciata*; *Aquatica lateralis*
**Glow Up! [4]**	Yes	Yes	No	No	Turin, Italy	*Luciola italica*
**Firefly Watch [5]**	No	Yes	Yes	Yes	North America	Adult flashing Lampyridae
**Has Visto Una Luciernaga? [6]**	Yes*	Yes	Yes	Yes**	Spain	Lampyridae
**Fireflyers International iNaturalist Project [7]**	Yes	Yes	Yes	No	Global	Lampyridae and other bioluminescent Coleoptera

**Clemson Disappearing Firefly Project [8]**	No	Yes	Yes	No	South Carolina, USA	Adult flashing Lampyridae
**Western Firefly Project [9]**	Yes*	Yes	Yes	Yes**	Western USA	Adult flashing Lampyridae
**Observatoire des Vers Luisants & des Lucioles [10]**	No	Yes	Yes*	No	France	Lampyridae
**Kreso Krijesnica [11]**	Yes*	Yes	Yes	Yes**	Croatia	Lampyridae
**Light Up West Virginia [12]**	Yes	Yes	Yes	No	West Virginia, USA	Adult flashing Lampyridae
**Grup Cucadellum [13]**	Yes*	Yes	Yes	Yes	Catalonia, Spain	Lampyridae
**Have you seen a firefly? (*ראיתם גחלילית?*) [14]**	Yes	Yes	Yes	No	Israel	Lampyridae
**Hong Kong Firefly Survey Team [15]**	Yes	Yes	Yes	Yes	Hong Kong, China	Lampyridae and Rhagophthalmidae

**Atlanta Firefly Project [16]**	Yes*	Yes	Yes	Yes	Georgia, USA	*Photinus pyralis* + other adult flashing Lampyridae
**World Firefly Day Count [17]**	No	Yes	Yes	Yes**	India	Adult flashing Lampyridae
**Magical Mysteries at Bukit Kiara [18]**	Yes	Unknown	Unknown	Unknown	Bukit Kiara Park, Kuala Lumpur, Malaysia	Lampyridae and Rhagophthalmidae
**Fireflies of Australia [19]**	Yes	Yes	Yes	No	Australia	Luciolinae
**Firefly Atlas [20]**	Yes	Yes	Yes	Yes	USA and Canada	Lampyridae
**Constructing a Colorado Firefly Flash Pattern Dataset [21]**	No*	Yes	Yes	Unknown	Colorado, USA	Adult flashing Lampyridae

1. *UK Glow worm survey home page*. https://www.glowworms.org.uk/ (accessed 12 September 2023).
2. *Glimwormenwerkgroep*. Natuurpunt. https://www.natuurpunt.be/afdelingen/glimwormenwerkgroep (accessed 12 September 2023).
3. Nature Conservation Society of Japan. *モニタリング1000里地調査： マニュアル・調査票 [Monitoring 1000 Satochi Survey: Manual/Survey Form]*. 日本自然保護協会オフィシャルサイト[Japan Nature Conservation Association official website]. https://www.nacsj.or.jp/activities/guardians/moni1000/howto/ (accessed 24 August 2023).
4. *Glow Up! Torino (2007) - Francesco Mariotti*. https://www.mariotti.ch/en/expositions/2007/glow/ (accessed 12 September 2023).
5. *Firefly Watch*. Mass Audubon. https://www.massaudubon.org/programs-events/community-science/firefly-watch (accessed 12 September 2023).

6. Guzmán Álvarez, J. R.; De Cock, R. *Gusanosdeluz | Luciérnagas. Esos bichitos mágicos.* https://www.gusanosdeluz.com/ (accessed 2023-09-28).
7. *Fireflyers International*. iNaturalist. https://www.inaturalist.org/projects/fireflyers-international (accessed 28 September 2023).
8. Chow, A.; Pargas, R. P.; Voges, A.; Li, B.; Edmonson, D.; Edison, G.; Hull, J.; Chong, J.-H.; Lyons, R. *Final Report: Clemson University's Vanishing Firefly Project: Using a Mobile Phone App as Educational and Research Tools for Sustainability*; EPA Grant Number: SU835496; U.S. Environmental Protection Agency. https://cfpub.epa.gov/ncer_abstracts//index.cfm (accessed 22 August 2023).
9. *Western Firefly Project: A Community Science Initiative | Natural History Museum of Utah*. https://nhmu.utah.edu/citizen-science/fireflies (accessed 12 September 2023).
10. *Observatoire des vers luisants*. http://www.asterella.eu/NEOKIPOS/formulaire0.php?pays=FRANCE&p= (accessed 28 September 2023).
11. *Krešo Krijesnica - Spasimo svjetla našeg djetinjstva*. https://www.facebook.com/kresokrijesnica (accessed 29 September 2023).
12. West Virginia Division of Natural Resources. *Light Up West Virginia's State Parks*. ArcGIS StoryMaps. https://storymaps.arcgis.com/stories/cc64f79fba9f41d2905aa6068ba13daa (accessed 28 September 2023).
13. *Grup Cucadellum (ICHN) – Grup d'estudi dels lampírids de Catalunya*. https://cucadellum.cat/ (accessed 12 September 2023).
14. *[Returning the light to the fireflies] מחזירים את האור לגחליליות*. החברה להגנת הטבע. https://www.teva.org.il/citizen-science/3391 (accessed 12 September 2023).
15. *Hong Kong Firefly Survey Team 香港螢火蟲調查隊*. HK Firefly Survey Team 香港螢火蟲調查隊. http://hkentsoc.org/hongkongfireflysurvey/hkentsoc.org/index.html (accessed 28 September 2023).
16. *Atlanta Firefly Project*. Atlanta Firefly Project. https://www.atlantafireflyproject.org (accessed 30 October 2023).
17. Rana, N.; Rayal, R.; Uniyal, V. P. Firefly Survey: Adopting Citizen Science Approach to Record the Status of Flashing Beetles. *Journal of Threatened Taxa* **2022**, *14* (10), 22016–22020. https://doi.org/10.11609/jott.7939.14.10.22016-22020.
18. *Magical Mysteries at Bukit Kiara*. Friends Of Bukit Kiara. https://www.fobk.org/fireflies/magical-mysteries-at-bukit-kiara/ (accessed 12 September 2023).
19. *Fireflies of Australia | Facebook*. https://www.facebook.com/groups/firefliesofaustralia/ (accessed 28 September 2023).
20. *Firefly Atlas | Home*. Firefly Atlas. https://www.fireflyatlas.org/ (accessed 28 September 2023).
21. *Constructing a Colorado Firefly Flash Pattern Dataset*. SciStarter. https://scistarter.org/constructing-a-colorado-firefly-flash-pattern-data (accessed 2023-09-28).
